# Impact of dietary purple neem (*Azadirachta indica*) leaves on growth, ability to neutralize free radicals and helminth control in meat goats

**DOI:** 10.3389/fvets.2026.1769618

**Published:** 2026-03-05

**Authors:** Pawinee Archa, Pornphutthachat Sota, Pramote Paengkoum

**Affiliations:** School of Animal Technology and Innovation, Institute of Agricultural Technology, Suranaree University of Technology, Nakhon Ratchasima, Thailand

**Keywords:** antioxidant, growth performance, nutrient intake, purple neem, strongyle-type

## Abstract

This study examined the effects of purple neem (PN) leaf supplementation in concentrate diets on growth performance, feed intake (FI), nutrient digestibility, antioxidant activity, and fecal parasite egg counts in crossbred male goats. Fifteen Chami × Anglo Nubian goats (20.43 ± 5.5 kg; aged 1–2 years) were randomly assigned to three treatments: 0, 3, and 6% PN leaf supplementation (DM basis). Goats were fed ad libitum with corn silage and concentrate diets containing 14% crude protein. PN supplementation significantly (*p* < 0.05) increased final body weight, weight gain, and dry matter (DM) intake, with the highest body weight at 6% inclusion. Digestibility of DM, crude protein (CP), and neutral detergent fiber (NDF) improved at 3 and 6% supplementation. Antioxidant activities (ABTS, DPPH, and SOD) were significantly enhanced, indicating improved antioxidant defense. Moreover, fecal strongyle egg counts decreased significantly in a dose-dependent manner, with the greatest reduction observed between days 28 and 56 at 3–6%. Overall, PN leaves demonstrate strong potential as a natural feed additive for improving health and productivity in goats.

## Introduction

Goat farming is an important economic activity in many regions of Thailand and is typically integrated with overall food production. Most smallholder farmers in Asia keep ruminants for labor, transport, manure, and to convert crop residues and forages into meat and milk, especially in tropical and subtropical areas (1). Goats are well adapted to diverse environmental conditions, making them suitable livestock for these regions (2). However, one of the main factors affecting goat productivity and growth is infection with gastrointestinal parasites (GIPs). Gastrointestinal nematodes (GINs) are particularly common in ruminants and pose a significant challenge, especially in grazing-based systems (3). Animals are exposed to infective larvae of parasites contaminating pastures, soil, and water sources. These parasites can damage the gastrointestinal mucosa, causing anemia, weakness, anorexia, diarrhea, weight loss, and reduced growth rates. Chronic parasitic infections can also suppress the immune system, increasing susceptibility to secondary infections. Ultimately, these impacts can lead to considerable losses in meat and milk production as well as decreased reproductive performance (4). The economic impact of parasitic infections in goats is therefore substantial, affecting both farms and the industry. Parasitism can reduce feed efficiency and extend the growth period, resulting in higher production costs. In many regions, cases of anthelmintic resistance have been reported due to the continued and improper use of deworming drugs, which further complicates the control and management of these infections (5).

Neem is a medicinal plant widely valued for its diverse therapeutic properties and broad-spectrum biological activities. Growing research interest in neem is driven primarily by its complex phytochemical profile, which includes high levels of bioactive compounds such as limonoids, tannins, flavonoids, alkaloids, saponins, and polyphenols (6). Many studies have shown that neem possesses antibacterial, antiviral, anti-inflammatory, antioxidanta and ntimalarial highlighting its potential as a natural remedy in various medical and veterinary fields (7). Additionally, the purple variety of neem has recently attracted attention due to its unique phytochemical profile and possible bioactivities, including compounds like anthocyanins. The wide range of pharmacological benefits and the safety profile of Neem (*Azadirachta indica*), which surpasses that of synthetic chemicals, has gained significant scientific interest. Furthermore, the anthelmintic mechanism of *A. indica* is believed to involve the synergistic effects of several bioactive compounds, such as azadirachtin, nimbin, and saponins (8). As a result, neem is recognized as a valuable natural resource with extensive potential in traditional medicine, sustainable agriculture, and the development of modern pharmaceutical products.

Previous studies have reported the use of PN in livestock nutrition. PN leaves and extracts have been shown to possess antioxidant, antimicrobial, and anthelmintic properties, and their inclusion in ruminant diets has been associated with reduced gastrointestinal parasite loads, improved rumen fermentation, and enhanced nutrient utilization when used at appropriate levels. However, information specifically on the use of PN leaves as a feed supplement in goats remains limited. Therefore, the present study was conducted to provide additional evidence on the effects of PN leaf supplementation on growth performance, antioxidant activity, and parasite control in goats (Table 1).

**Table 1:** Ingredients and chemical composition of experimental diets.

Items	Ingredients		
	Corn silage	Concentrate	PN^1^
Composition, % of DM			
DM^2^	35.64	88.33	41.55
Ash	6.69	2.16	3.53
CP^3^	5.56	14.04	3.16
EE^4^	3.69	1.11	5.32
NDF^5^	50.14	7.59	12.49
ADF^6^	28.45	5.32	10.81

^1^PN, purple neem; ^2^DM, dry matter; ^3^CP, crude protein; ^4^EE, ether extract; ^5^NDF, neutral detergent fiber; ^6^ADF, acid detergent fiber.

## Methods

### Ethical approval

This study was approved by the Institute of Research and Development (IRD), Suranaree University of Technology (SUT) (IACUC-67-31) and Biosafety SUT (BIC-67-24).

### Animals, diets, and experimental design

Fifteen female meat goats (Chami x Anglo Nubian) weighing 20.43 ± 5.5 kg and between the ages of 1 to 2 years, the animals were randomly assigned to three experimental groups, each with five replications, following a completely randomized design (CRD). All these goats were held individually in cages (1.5 m × 1.5 m) equipped with feeders and water. The experimental conditions for a period of 10 days before the commencement of the trial. During both the adaptation (14 days), and experimental phases (90 days), the animals were fed twice daily at 6:30 a.m. and 4:30 p.m. and provided with clean feed and water troughs.

Fresh NP leaves were oven-dried at 60 °C to a constant weight and crushed through a 1 mm sieve. For nutritional composition analysis, DM, CP, EE, NDF, and ADF. All treatment groups received corn silage as the basal roughage *ad libitum*, along with varying levels of PN supplementation as follows: T1 = control (0%), T2 = 3%, and T3 = 6% on a DM basis. Additionally, all goats were supplemented with a commercially formulated concentrate pellet containing 14% crude protein. The experimental diet before and after 21 days. The objective of this experiment was to evaluate the effects of supplementing PN (leaf) on GIPs in meat goats at 0, 14, 28, and 56 days. The experimental goats were naturally infected with parasites prior to the study.

### Nutrient intakes

Feed intake (FI) was recorded daily by subtracting refusals from the amount offered.

Fecal samples were collected during the last week of each period for chemical analyses following the AOAC (9) method. Nutrient intakes (DM, CP, EE, NDF, and ADF) were calculated by subtracting the nutrient content of feed refusals from that of the feed offered. Apparent nutrient digestibility was determined during the last week of each period. Body weights of the meat goats were recorded on the first and last sampling days of each period. Feces were collected before feeding, and digestibility was calculated as the proportion of ingested nutrients absorbed by the animal rather than excreted, using the formula: Digestibility (%) = [(dietary intake−feces output)/dietary intake] × 100. Body weights of the meat goats were recorded on the first and last sampling days of each period.

### Stool collection and processing

Fresh fecal samples were collected individually from goats housed in separate raised cages equipped with nets beneath to prevent contamination (fecal samples were collected directly from the rectum at each sampling). Sampling was performed in the morning (06:00 a.m.) by collecting a fresh fecal sample from each net using sterile clean gloves. Approximately 5 g of feces per goat was placed in sterile plastic bags. Samples were kept in a cooler at 4 °C and transported to the laboratory SUT, within 1 h and continued processing using the Modified Formalin–Ether Concentration Technique (M-FECT), modified from standard protocols (10, 11). Briefly, approximately 2 g of feces was mixed with 10 mL of 10% formalin and homogenized until it was suspended. The suspension was filtered through two layers of wet cotton gauze, and the filtrate volume was adjusted to 10 mL with an additional 10% formalin. Subsequently, samples were centrifuged at 1,500 revolutions per minute for 3 min. Following centrifugation, the supernatant was discarded, and the residual pellet was resuspended in 7 mL of 10% formalin and 3 mL of ethyl acetate. The mixture was shaken vigorously for 30 s to 1 min before the subsequent stage, which consisted of centrifugation at the same speed and duration. The upper layers were carefully discarded, retaining approximately 0.5 mL of formalin and the sediment at the bottom. The sediment was resuspended; a few drops were placed on a glass slide, covered with a coverslip, and examined microscopically at 400x magnification. Parasite eggs and cysts were morphologically identified in accordance with Taylor et al. (12). The degree of infection was measured as eggs per gramme of feces (EPG).

At the end of each period, blood samples (5 mL) were collected at 0, 2, and 4 h post-feeding for the determination of antioxidant activity using DPPH and ABTS assays according to Dawidowicz et al. (13), SOD assays according to Sirota (14). and readings were obtained using a Multiskan GO spectrophotometer with analysis performed in SkanIt 4.1 software.

Analysis of variance was used in the statistical analysis which was carried out using the Statistical Package for Social Science (SPSS) 25 program (27). Mean intensity of egg emission was calculated as the arithmetic mean of the EPG only of the infected animals. Confidence level was held at 95%, and *p* < 0.05 was set for significance; all analyses were undertaken in SPSS 25 program.

## Results

Supplementation of PN leaves in concentrate diets at 3 and 6% significantly (*p* < 0.05) improved the growth performance, FI, and nutrient digestibility of goats. The final live weight and weight gain were highest in goats receiving 6% PN supplementation (46.81 kg), and DM intake (DMI, g/kg BW^0.75) also increased significantly (*p* < 0.05) compared with the control group. DM, CP, EE, NDF, and acid detergent fiber (ADF) intakes increased linearly (*p* < 0.05) with increasing PN levels in the concentrate.

Goats receiving 6% PN showed the highest intake values among all groups. For nutrient digestibility, supplementation with PN leaves significantly (*p* < 0.05) enhanced the digestibility of DM, CP, and NDF, with goats in the 3 and 6% groups exhibiting higher digestibility than those in the control group (DM: 50.89 and 50.71% vs. 36.39%; CP: 54.48 and 54.44% vs. 39.74%; NDF: 73.03 and 73.95% vs. 60.98%). In contrast, EE digestibility showed a decreasing linear trend (*p* < 0.05), whereas ADF digestibility did not differ among the treatments (*p* > 0.05).

PN supplementation also enhanced antioxidant activity, as indicated by increases in ABTS (mg/ml), DPPH (mg/ml), and SOD (U/ml) values. At 0 h, no significant differences (*p* > 0.05) were observed among the treatments; however, after 3 and 6 h of incubation, antioxidant activities increased significantly (*p* < 0.05), indicating that PN possesses strong potential to enhance the antioxidant defense system in animals.

**Figure 1:**
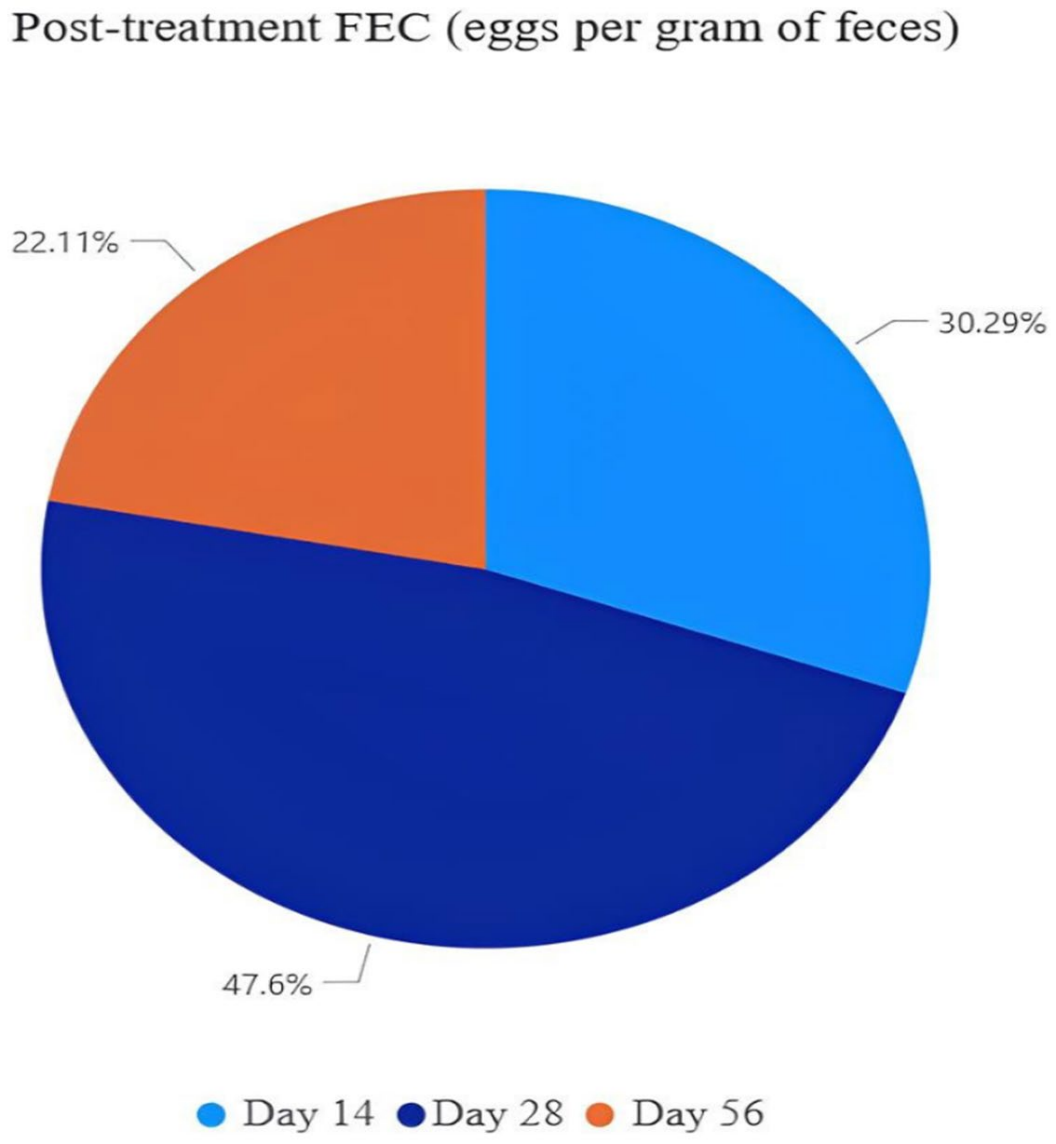
Effect of PN at three levels: T1 = 0% (control), T2 = 3%, and T3 = 6% (on a DM basis), with each diet containing 14% crude protein on EPG of strongyle-type and post-treatment FEC (eggs per gram of feces) in meat goats.

Furthermore, this observation aligns with the present study, in which supplementation with PN leaves significantly (*p* < 0.05) reduced the fecal egg count (FEC) of strongyle-type parasites (Table 2). A dose-dependent reduction was observed, particularly in goats supplemented with 3–6% PN leaves between 28 and 56 days. From the pie chart (Figure 1), it is evident that the FEC following treatment showed a continuous decreasing trend over time. The proportion of FEC was 30.29% on day 14, 47.60% on day 28, and 22.11% on day 56. These results indicate that the treatment was effective in reducing the number of parasite eggs over time.

**Table 2:** Effect of PN on EPG of Strongyle-type in meat goats 0,14,28,56 day.

Item	levels of PN in concentrate (%)			SEM^1^	*p*-value^2^		
	0	3	6		Linear	Quadratic	Cubic
Strongyle-type							
0 day	150.40	179.50	434.20^a^	60.25	*	0.34	0.34
14 day	508.80	683.00	486.110	104.22	0.93	0.44	0.44
28 day	1055.20^a^	114.20^c^	267.50^b^	194.96	*	*	0.58
56 day	1174.20^a^	19.80^c^	30.60^b^	216.55	*	*	0.14

^abc^ Mean on the same row having different superscripts were significantly different *: significantly different, **: highly significantly different,^1^standard error of mean, ^2^probability value different.

## Discussion

Supplementation of PN leaves in concentrate diets at levels of 0, 3, and 6% significantly (*p* < 0.05) (Table 3) improved the growth performance, FI, and nutrient digestibility of goats. Goats receiving 6% PN supplementation exhibited the highest final body weight and weight gain (46.81 kg), along with a significantly higher DM intake (DMI, g/kg BW^0.75) than that of the control group (0%). These findings indicate that PN leaf supplementation stimulated FI and enhanced nutrient utilization efficiency in broiler chickens. Furthermore, the intakes of DM, CP, EE, NDF, and ADF increased linearly (*p* < 0.05) with increasing levels of PN in the concentrate, suggesting that higher inclusion levels promoted better nutrient intake and utilization efficiency in goats.

**Table 3:** Effect of PN on nutrient intake, digestibility, and growth performance by the meat goats when fed the experimental rations.

Items	PN level in concentrate (%)			SEM^1^	*p*-value^2^		
	0	3	6		Linear	Quadratic	Cubic
Initial weight, kg	25.88	25.70	25.08	2.47	0.83	0.65	0.65
Final live weight	32.08^c^	35.47^b^	41.44^a^	2.39	*	0.35	0.35
Live weight gain	32.62^c^	46.10^b^	46.81^a^	2.10	*	0.45	0.45
Weight change, g/d	5.03	4.12	5.23	0.23	0.70	0.45	0.45
DMI, g/kg BW^0.75^	16.27^c^	18.79^b^	19.45^a^	0.67	*	0.61	0.49
Nutrient intake, g/d							
DM^3^	1809.53^c^	2014.23^b^	2574.28^a^	17.89	*	0.43	0.43
CP^4^	116.77^c^	129.88^b^	166.40^a^	7.94	*	0.38	0.38
EE^5^	143.09^c^	161.54^b^	180.00^a^	4.02	*	0.55	0.55
NDF^6^	915.18^c^	1047.11^b^	1389.38^a^	73.11	*	0.37	0.37
ADF^7^	835.57^c^	800.01^b^	955.56^a^	26.01	*	0.48	0.48
Digestibility, %							
DM	36.39^c^	50.89^ab^	50.71^b^	4.27	*	0.42	0.42
CP	39.74^c^	54.48^ab^	54.44^b^	4.37	*	0.43	0.43
EE	30.18^c^	22.21^ab^	22.20^b^	1.96	*	0.40	0.40
NDF	60.98^c^	73.03^b^	73.95^ab^	2.41	*	0.45	0.44
ADF	80.49	81.99	80.72	1.03	0.93	0.56	0.56

^abc^ Mean on the same row having different superscripts were significantly different *: significantly different, **: highly significantly different, ^1^standard error of mean; ^2^probability value different; ^3^DM, dry matter; ^4^CP, crude protein, ^5^EE, ether extract; ^6^NDF, neutral detergent fiber; ^7^ADF, acid detergent fiber.

This may be attributed to the fact that the inclusion of PN leaves enhanced diet palatability, leading to increased FI by goats. In addition, the bioactive compounds present in PN leaves, such as flavonoids and anthocyanins, possess antioxidant properties that can reduce oxidative stress and improve rumen health, thereby enhancing digestion and nutrient absorption efficiency (15). Moreover, low levels of phenolic compounds and tannins may help modulate the rumen microbial balance by promoting the growth of fiber-digesting microorganisms, which consequently improves nutrient utilization efficiency (16).

Therefore, the supplementation of PN leaves in concentrate diets has the potential to stimulate FI and improve nutrient utilization efficiency in goats. The observed improvement may be attributed to the presence of bioactive compounds in PN leaves, such as flavonoids and anthocyanins, which possess antioxidant properties that help reduce oxidative stress, thereby enhancing rumen health and fermentation efficiency (17). This may lead to improved digestion and nutrient absorption in the host. In addition, the inclusion of PN leaves may have enhanced diet palatability, resulting in greater FI without adverse effects on overall health. These results suggest that supplementation of PN leaves in concentrate diets, particularly at the 6% level (Table 3), has the potential to effectively improve growth performance and feed utilization efficiency in goats. These results support the findings of Kholif and Olafadehan (18). It has been reported that supplementation of herbal plants containing low levels of polyphenols and tannins can enhance the digestibility of protein and fiber in ruminants. These compounds help reduce ruminal protein degradation and promote greater absorption of undegraded proteins in the small intestine. Simultaneously, they contribute to maintaining a balanced rumen microbial ecosystem that favors fiber fermentation, thereby improving the overall efficiency of nutrient utilization in the host.

In terms of nutrient digestibility, supplementation with neem leaves at 3 and 6% significantly (*p* < 0.05) (Table 3) increased the digestibility of DM, CP, and NDF compared to the control group. The DM, CP, and NDF digestibility values of the supplemented groups ranged from 50.71 to 54.48%, which were higher than those of the control group (36.39–39.74%); these results suggest that neem leaves positively influence rumen microflora activity, particularly cellulose- and hemicellulose-degrading bacteria, which may benefit from the reduced accumulation of excess ammonia and the inhibition of methane-producing microorganisms in the fermentation system. However, EE digestibility tended to decrease with increasing PN leaf supplementation, which may be attributed to the chemical composition of the feed and tannins capable of binding to fat molecules, thereby slightly reducing fat digestibility. In contrast, ADF digestibility did not differ significantly (*p* > 0.05) (Table 3) among the experimental groups, indicating that supplementation with PN leaves at the studied levels did not adversely affect structural fiber digestion. These findings are consistent with those of Wanapat et al. (19), who reported that supplementation with certain herbal plants, such as garlic, Indian gooseberry, and neem, at low levels can enhance FI and improve digestibility in goats and beef cattle.

Additionally, supplementation with PN leaves markedly enhanced antioxidant activity, as evidenced by significantly higher ABTS, DPPH, and SOD enzyme activities (*p* < 0.05) after 3 and 6 h of incubation compared with the control group (Table 4). This indicates that PN leaves have considerable potential to promote antioxidant defense in animals. These results are in line with those of Islas et al. (20), who reported that neem leaf extracts contain natural antioxidants, such as flavonoids, catechins, and polyphenols, which help prevent cellular damage by free radicals and reduce oxidative stress in ruminants. The inclusion of these antioxidants can contribute to maintaining the immune system balance and promoting overall animal health. PN leaves contain various phytochemicals, including phenolics, flavonoids, and tannins, which exhibit direct antioxidant activities. These compounds can donate electrons or reduce the oxidative reactions of free radicals, thereby mitigating cellular and tissue damage (20). Additionally, Tannins are polyphenolic compounds that, upon ingestion by animals, form tannin–protein complexes that exhibit alkaline properties as they pass into the small intestine. This process increases intestinal pH, creating an environment unfavorable for parasite survival, thereby inhibiting the development of parasitic larvae (21). Supplementation with PN leaves in animal diets or biological systems can stimulate the body to enhance the activity of key antioxidant enzymes, such as: ABTS is used to evaluate the ability of compounds to scavenge ABTS^+^ radicals (22). DPPH is used to assess the electron-donating ability of compounds that scavenge DPPH radicals (23). SOD is a crucial enzyme that catalyzes the dismutation of superoxide radicals (O₂^−^•) into molecular oxygen and hydrogen peroxide, and represents the first line of defense against oxidative stress in cells (24). Therefore, supplementation with PN leaves enhances the efficiency of the antioxidant defense system both directly (through bioactive compounds present in the leaves) and indirectly (by stimulating endogenous antioxidant enzymes), resulting in a significant increase in ABTS, DPPH, and SOD activities.

**Table 4:** Effect of PN on antioxidant activity by the meat goats when fed the experimental rations.

Items	PN level in concentrate (%)			SEM^1^	*p*-value^2^		
	0	3	6		Linear	Quadratic	Cubic
Antioxidant activity							
*ABTS (mg/ml)* ^3^							
0 h	23.60	25.20	25.30	0.82	0.43	0.68	0.68
3 h	34.00^c^	47.40^b^	53.30^a^	2.32	*	0.81	0.81
6 h	52.50^c^	74.10^a^	74.00^b^	2.77	*	*	*
*DPPH (mg/ml)* ^4^							
0 h	53.30	41.42	50.30	1.69	0.70	0.15	0.15
3 h	53.62	57.44	54.84	0.91	0.79	0.43	0.43
6 h	54.80^c^	65.40^b^	63.60^a^	1.41	*	*	*
*SOD (U/ml)* ^5^							
0 h	24.90	25.50	24.40	0.61	0.75	0.55	0.55
3 h	45.30^c^	65.10^b^	60.90^a^	2.36	*	*	*
6 h	50.70^c^	70.50^b^	76.20^a^	3.19	*	0.34	0.34

^abc^ Mean on the same row having different superscripts were significantly different *: significantly different, **: highly significantly different, ^1^standard error of mean; ^2^probability value different; ^3^ABTS, (2,2′-azinobis-(3-ethylbenzothiazoline-6-sulfonic acid)); ^4^DPPH, (2,2-diphenyl-1-picrylhydrazyl); SOD, Superoxide Dismutase.

**Figure 2:**
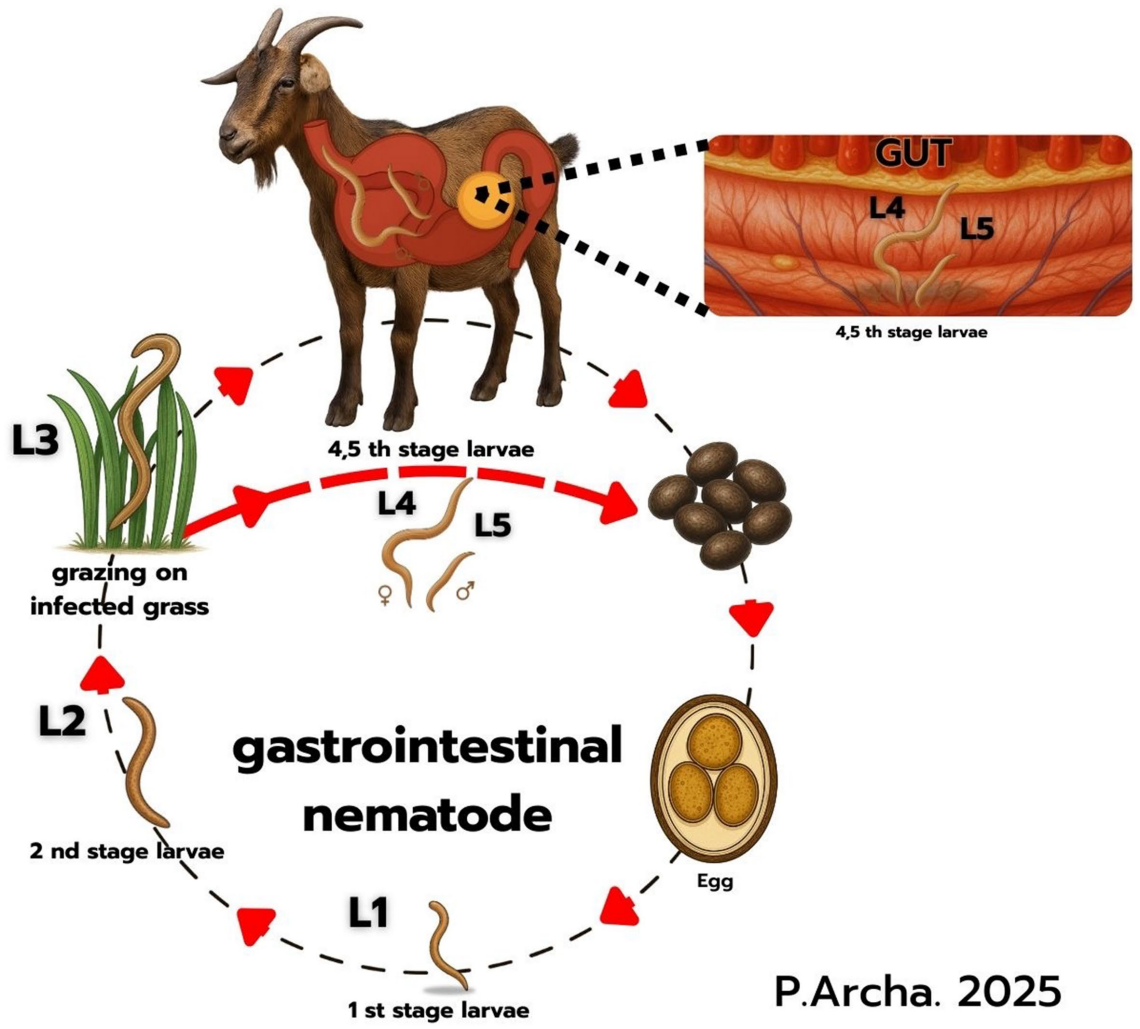
The life cycle of Strongyle-type parasites (*Strongylidae*) involves both environmental and host stages and commonly affects ruminants such as goats, sheep, and cattle. Eggs excreted in feces hatch into larvae (L1–L3) under suitable conditions, with L3 being the infective stage that migrates onto herbage and is ingested by animals. Inside the host, larvae develop into adults (L4–L5) in the intestines, where they feed on blood or tissue, causing anemia, diarrhea, weight loss, and reduced productivity. Mature worms lay eggs that are passed in feces, completing the cycle.

Gastrointestinal nematodes are important internal parasites that significantly affect the health and productivity of animals. These parasites can cause anemia, reduced growth rates, weight loss, and decreased production (25). Infection in goats commonly occurs through the ingestion of infective larvae contaminating pastures or water sources (Figure 2). Previous studies have suggested a potential anthelmintic effect of neem leaves in the gastrointestinal tract, which is consistent with the findings of Rahaman et al. (26). This observation aligns with the present study, in which supplementation with PN leaves significantly (*p* < 0.05) reduced the fecal egg count (FEC) of strongyle-type parasites (Table 2). A dose-dependent reduction was observed, particularly in goats supplemented with 3–6% PN leaves between 28 and 56 d. From the pie chart (Figure 1), it is evident that the FEC following treatment showed a continuous decreasing trend. The proportion of FEC was 30.29% on day 14, 47.60% on day 28, and 22.11% on day 56. These results indicate that the treatment was effective in reducing the number of parasite eggs.

The anthelmintic mechanism of neem may be associated with the synergistic action of several bioactive compounds, such as azadirachtin, nimbin, and saponins (8). These compounds are known to interfere with collagen synthesis and the formation of the parasite’s external structure and inhibit the embryonic development of parasite eggs, preventing maturation into larvae. Moreover, the reduction in the gastrointestinal parasite burden contributes to improved health status, growth performance, and nutrient utilization efficiency in goats. The findings of this study indicate that supplementing goat concentrate diets with 3–6% PN leaves effectively promotes growth, enhances feed utilization efficiency, strengthens antioxidant defense mechanisms, and significantly reduces gastrointestinal parasitic infections in goats. Therefore, PN leaves hold considerable potential as a safe natural feed additive capable of reducing the reliance on chemical anthelmintics and supporting sustainable ruminant production systems in the future.

## Conclusion

Supplementation of PN leaves in concentrate diets at 6% improved growth performance, FI, and nutrient digestibility of goats and enhanced antioxidant activity. Furthermore, PN has the capacity to control nematodes in goats, with the highest reduction observed between days 28 and 56 when supplemented at 3–6%. This finding reveals that PN could be considered for a natural feed additive with strong antioxidant and anthelmintic properties that promote ruminant health and production.

## Data Availability

The original contributions presented in the study are included in the article/supplementary material, further inquiries can be directed to the corresponding author.
